# Surface Hopping Dynamics on Vibronic Coupling Models

**DOI:** 10.1021/acs.accounts.1c00485

**Published:** 2021-09-27

**Authors:** J. Patrick Zobel, Moritz Heindl, Felix Plasser, Sebastian Mai, Leticia González

**Affiliations:** † Institute of Theoretical Chemistry, Faculty of Chemistry, University of Vienna, Währingerstr. 19, 1090 Vienna, Austria; ‡ Department of Chemistry, Loughborough University, Loughborough LE11 3TU, United Kingdom; § Vienna Research Platform on Accelerating Photoreaction Discovery, University of Vienna, Währingerstr. 19, 1090 Vienna, Austria

## Abstract

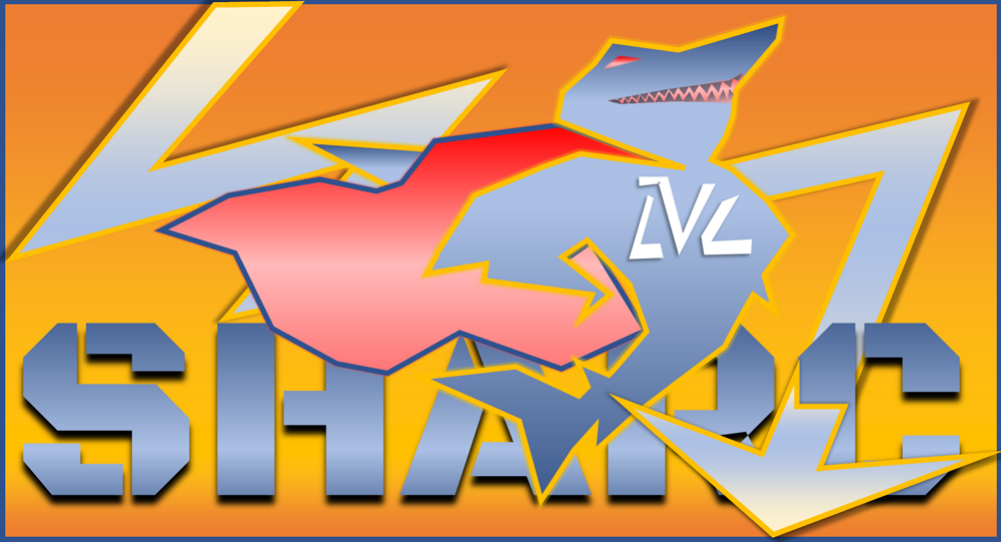

The simulation of photoinduced non-adiabatic dynamics is of great relevance in many scientific disciplines, ranging from physics and materials science to chemistry and biology. Upon light irradiation, different relaxation processes take place in which electronic and nuclear motion are intimately coupled. These are best described by the time-dependent molecular Schrödinger equation, but its solution poses fundamental practical challenges to contemporary theoretical chemistry. Two widely used and complementary approaches to this problem are multiconfigurational time-dependent Hartree (MCTDH) and trajectory surface hopping (SH). MCTDH is an accurate fully quantum-mechanical technique but often is feasible only in reduced dimensionality, in combination with approximate vibronic coupling (VC) Hamiltonians, or both (i.e., reduced-dimensional VC potentials). In contrast, SH is a quantum–classical technique that neglects most nuclear quantum effects but allows nuclear dynamics in full dimensionality by calculating potential energy surfaces on the fly. If nuclear quantum effects do not play a central role and a linear VC (LVC) Hamiltonian is appropriate—e.g., for stiff molecules that generally keep their conformation in the excited state—then it seems advantageous to combine the efficient LVC and SH techniques. In this Account, we describe how surface hopping based on an LVC Hamiltonian (SH/LVC)—as recently implemented in the SHARC surface hopping package—can provide an economical and automated approach to simulate non-adiabatic dynamics. First, we illustrate the potential of SH/LVC in a number of showcases, including intersystem crossing in SO_2_, intra-Rydberg dynamics in acetone, and several photophysical studies on large transition-metal complexes, which would be much more demanding or impossible to perform with other methods. While all of the applications provide very useful insights into light-induced phenomena, they also hint at difficulties faced by the SH/LVC methodology that need to be addressed in the future. Second, we contend that the SH/LVC approach can be useful to benchmark SH itself. By the use of the same (LVC) potentials as MCTDH calculations have employed for decades and by relying on the efficiency of SH/LVC, it is possible to directly compare multiple SH test calculations with a MCTDH reference and ponder the accuracy of various correction algorithms behind the SH methodology, such as decoherence corrections or momentum rescaling schemes. Third, we demonstrate how the efficiency of SH/LVC can also be exploited to identify essential nuclear and electronic degrees of freedom to be employed in more accurate MCTDH calculations. Lastly, we show that SH/LVC is able to advance the development of SH protocols that can describe nuclear dynamics including explicit laser fields—a very challenging endeavor for trajectory-based schemes. To end, this Account compiles the typical costs of contemporary SH simulations, evidencing the great advantages of using parametrized potentials. The LVC model is a sleeping beauty that, kissed by SH, is fueling the field of excited-state molecular dynamics. We hope that this Account will stimulate future research in this direction, leveraging the advantages of the SH/VC schemes to larger extents and extending their applicability to uncharted territories.

## Key References

- PlasserF.; GómezS.; MengerM. F. S. J.; MaiS.; GonzálezL.Highly efficient surface hopping dynamics using a linear vibronic coupling model. Phys. Chem. Chem. Phys.2019, 21, 57–6910.1039/c8cp05662e30306987.^1^*This work describes a general, computationally efficient, and user-friendly implementation of surface hopping (SH) using linear vibronic coupling (LVC)-parametrized potential energy surfaces.*
- ZobelJ. P.; KnollT.; GonzálezL.Ultrafast and long-time excited-state kinetics of an NIR emissive vanadium(III) complex. II. Elucidating triplet-to-singlet excited-state dynamics. Chem. Sci.2021, 12, 10791–108013447606010.1039/d1sc02149dPMC8372553.^2^*This work showcases SH/LVC simulations of an open-shell metal complex with a degenerate triplet ground state for several picoseconds in full dimensionality.*
- PlasserF.; MaiS.; FumanalM.; GindenspergerE.; ChantalD.; GonzálezL.Strong influence of decoherence corrections and momentum rescaling in surface hopping dynamics of transition metal complexes. J. Chem. Theory Comput.2019, 15, 5031–5045.3133971610.1021/acs.jctc.9b00525(3)*This work illustrates how SH/LVC allows investigation of surface hopping parameters in a one-to-one comparison with high-level quantum dynamics methods.*
- GómezS.; HeindlM.; SzabadiA.; GonzálezL.From Surface Hopping to Quantum Dynamics and Back. Finding Essential Electronic and Nuclear Degrees of Freedom and Optimal Surface Hopping Parameters. J. Phys. Chem. A2019, 123, 8321–83323147926510.1021/acs.jpca.9b06103.^4^*The iterative SHARC-gym algorithm is introduced as a gateway to find reduced LVC models of complex systems.*

## 1 Introduction

**Figure 1:**
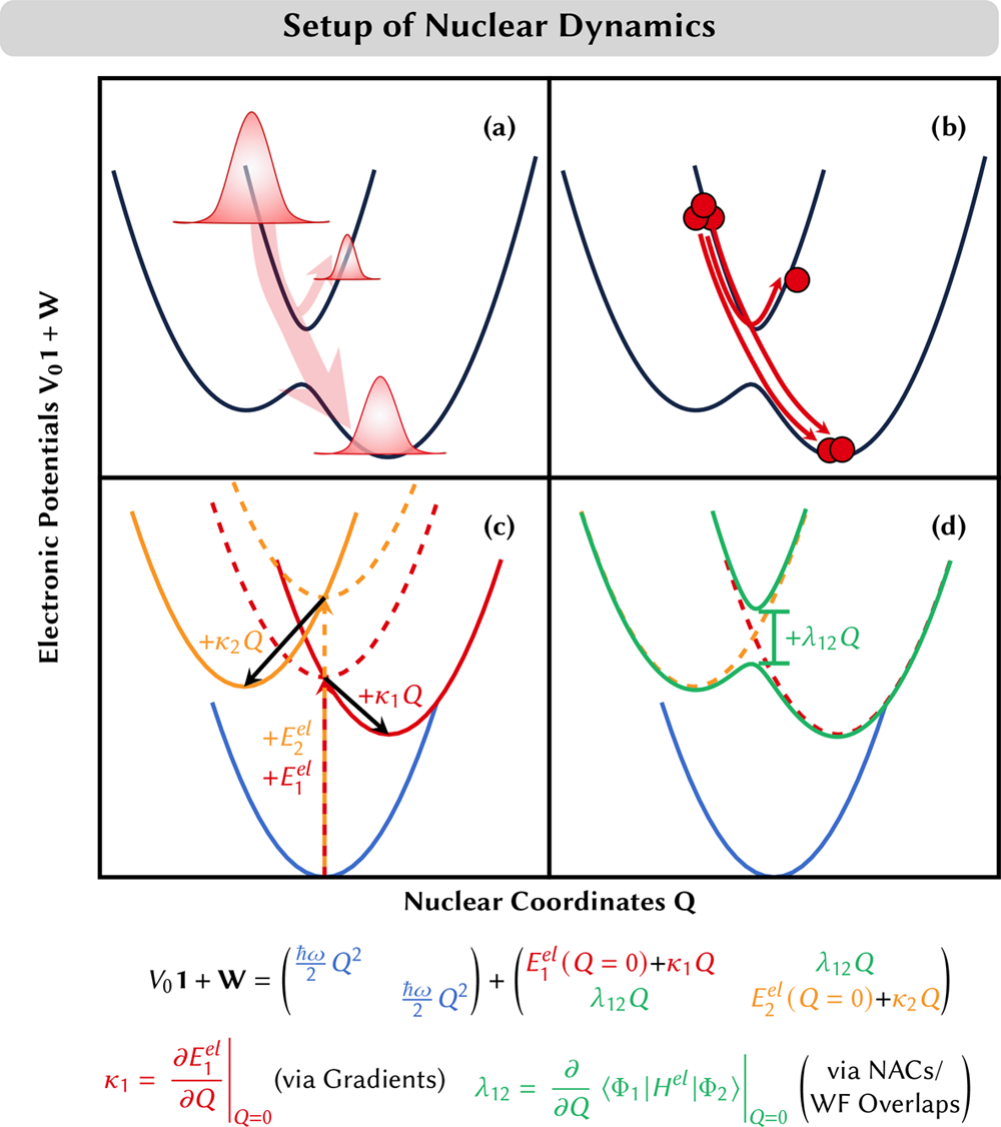
(a, b) Schematics of the behaviors of wave packet dynamics and SH dynamics, respectively. (c, d) Construction of LVC potentials. Intrastate couplings κ and interstate couplings λ can be calculated via gradients and non-adiabatic couplings or wave function overlaps, respectively. Adapted from ref (21). CC BY 4.0.

Photoinduced processes play an essential role in many scientific fields. The involved coupled electronic–nuclear motion can be described with the time-dependent Schrödinger equation—typically, solving the electronic and nuclear problems sequentially. First, the electronic Schrödinger equation delivers potential energy surfaces (PESs) and, for coupled electronic states, non-adiabatic couplings (NACs). The subsequent propagation of a wave packet (Figure 1a) requires solving the nuclear Schrödinger equation on the entire multidimensional PESs, which are traditionally represented as discretized grids whose size grows exponentially with the number of nuclear degrees of freedom (DOF).^5^

One avenue to evade this exponential growth—known as the curse of dimensionality—is to construct analytical, parametrized models of the PESs. A well-known recipe is vibronic coupling (VC) theory.^6,7^ By construction, VC models reproduce the correct shape of conical intersections^8^ and thus capture the essential physics of non-adiabatic transitions in a compact form (including S_0_/S_1_ conical intersections when a suitable quantum-chemical method is used for parametrization). Spin–vibronic coupling models can be used when different spin multiplicities are involved.^9^ Besides computational efficiency, VC models are attractive because of their conceptual simplicity and the low effort required to parametrize high-dimensional PESs from a few electronic structure calculations.^1,10^ These features distinguish them from related approaches, such as machine learning^11,12^ or interpolation of diabatic Hamiltonians,^13^ which can potentially deliver more accurate PESs but currently are more difficult to use. For these reasons, VC models have been extensively used in combination with the multiconfigurational time-dependent Hartree (MCTDH) method^14−16^ for quantum dynamics (QD) studies and to obtain vibronic spectra of moderately sized stiff molecules.^7,9,15,17^ In recent years, VC models experienced a renaissance in the field of exciton dynamics^18,19^ and in the context of the Frenkel–Holstein vibronic exciton Hamiltonian.^20^ However, MCTDH dynamics with VC potentials still formally scales exponentially, becoming infeasible for large systems.

Besides other developed QD schemes,^22^ non-adiabatic dynamics can be simulated with independent trajectory surface hopping (SH) methods.^23^ In SH, nuclei are described with a swarm of independent classical trajectories that follow the gradients of their active PES and switch the active surface—called a “surface hop”—if the electronic couplings indicate a non-adiabatic transition (Figure 1b). Within the herein-employed classical approximation featuring noninteracting trajectories, some nuclear quantum effects—such as tunneling, zero-point energy, or interferences—cannot be described properly. However, SH has several practical advantages: (i) it allows “on the fly” simulations in which electronic structure properties are calculated only for actually visited geometries; (ii) it is intuitive to interpret; and (iii) it has favorable computational scaling. Thus, SH can formally deal with systems of arbitrary size, limited only by the cost of the underlying electronic structure calculations at each time step. This cost can be very much alleviated if SH is combined with VC model potentials.

Extending early work,^24^ in 2019 we implemented a general linear VC (LVC) model^1^ and automatic parametrization routines^25^ in the SH program package SHARC.^26,27^ This SH/LVC “sleeping beauty” has awakened dynamical studies of systems with a few hundreds of DOFs including thousands of trajectories and extending to multiple picoseconds. The combined SH/LVC approach has also eased the benchmarking of SH protocols against more accurate quantum methods. Here, after reviewing the basic theory behind VC (section 2), we illustrate the capabilities of SH/LVC with several applications (sections 3 and 4) and its limitations.

## 2 Vibronic Coupling Theory

Within VC theory,^6^ the PESs are described through the general Hamiltonian^28^
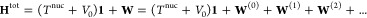
1where the ground-state potential *V*_0_ is usually approximated by harmonic oscillators in normal mode coordinates *Q*:
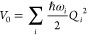
2The potential energy matrix **W** is expanded in a Taylor series around the reference geometry *Q*_0_, and its elements are written in a basis of diabatic states *m*, *n* as
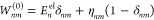
3
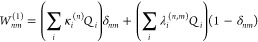
4
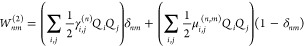
5The zeroth-order terms in eq 3 correspond to vertical excitation energies *E*_*n*_^el^ and optional constant coupling terms η_*nm*_ that are used to introduce weakly geometry-dependent couplings like spin–orbit couplings^25,28,29^ or excitonic couplings.^18,19^ The first-order terms in eq 4 define the widely used LVC model,^28,30−32^ including the gradient-like intrastate couplings κ_*i*_^(*n*)^ and the linear interstate coupling terms λ_*i*_^(*n,m*)^ that mediate interactions between the diabatic states. The construction of an LVC model, including the effect of the first-order parameters, is sketched in Figure 1c,d.

The potentials can be easily parametrized from a single-point calculation including gradients and NACs.^1,10^ If NACs are not available, the parameters can be obtained using finite differences (at a minimum of ∼6*N*_atom_ single-point calculations), given that the potentials can be diabatized, e.g., using wave function overlaps.^25,33,34^ The parametrization is performed in the diabatic basis, as diabatic PESs are smoother than adiabatic PESs and thus easier to parametrize. The PESs can then be transformed into the adiabatic basis to perform SH dynamics, or one can use the diabatic PESs to perform QD, e.g., using the MCTDH method.

Further flexibility can be obtained by including second-order terms^35,36^ (eq 5), which is known as quadratic vibronic coupling (QVC). Because parametrizing QVC or higher-order PESs requires more points per mode, most applications—including the ones described in the following—rely on LVC models. We note that dynamics based on VC potentials is still restricted to exploration of the PES close to the reference geometry. Photochemical reactions that lead to different conformers in the ground-state PES cannot be described. Similarly, certain motions such as torsion or dissociation cannot be described by simple VC models; it is then necessary to introduce further specifically designed potentials.

## 3 Applications of Surface-Hopping Dynamics with LVC Models

### 3.1 Sulfur Dioxide Intersystem Crossing

**Figure 2:**
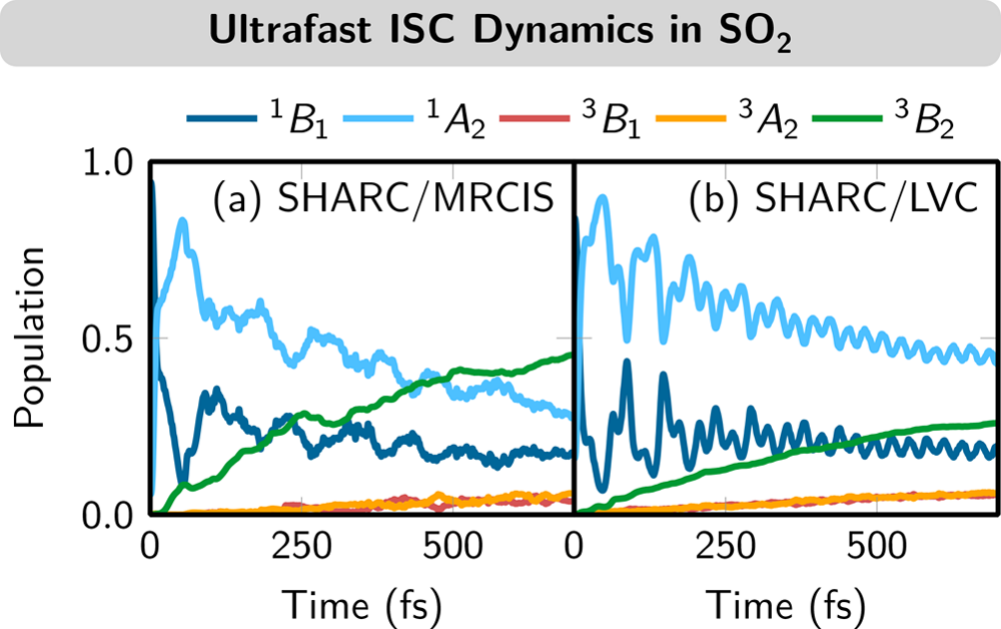
Diabatic populations of SO_2_ during ISC dynamics. (a) Results using ab initio SH (MRCIS). Data were taken from ref (37). (b) Results using an LVC model (parametrized on MRCIS).

Although it is small, sulfur dioxide (SO_2_) exhibits fascinating photophysics that remained elusive for decades. Our 2014 dynamical study,^37^ based on multireference configuration interaction with single excitations (MRCIS) including spin–orbit couplings,^38^ clarified that SO_2_ undergoes ultrafast (subpicosecond) intersystem crossing (ISC) mainly to one of the three triplet states available in the relevant energy range (^3^B_2_), as shown in Figure 2a. These simulations required a notable amount of CPU time (∼15 000 CPUh). SO_2_ was thus an appropriate test bed to find out whether the SH/LVC approach was able to return the correct photophysical behavior at a fraction of the computational cost.

To this aim, an LVC model was parametrized from a single MRCIS calculation using a “one-shot” approach.^1^ The resulting population dynamics (Figure 2b) demonstrates that the simple LVC model is able to qualitatively reproduce the main features of the ab initio results: initial population transfer from ^1^B_1_ to ^1^A_2_, long-lived oscillations between those two states, and ultrafast ISC to the ^3^B_2_ state. This is very encouraging given that the primary triplet state(s) involved in the ISC process were heavily disputed in the past.^39^ The main limitation of the employed LVC model of SO_2_ is that the ground-state harmonic oscillator in *V*_0_ is too stiff to describe the excited states, explaining the too-fast oscillations in Figure 2b. Overall, 1800 trajectories (amounting to 1.26 ns of total simulation time) were propagated using the efficient “pysharc” implementation with optimized file I/O^1,29^ at a total cost of merely 5 CPUh.

### 3.2 Acetone Intra-Rydberg-State Dynamics

**Figure 3:**
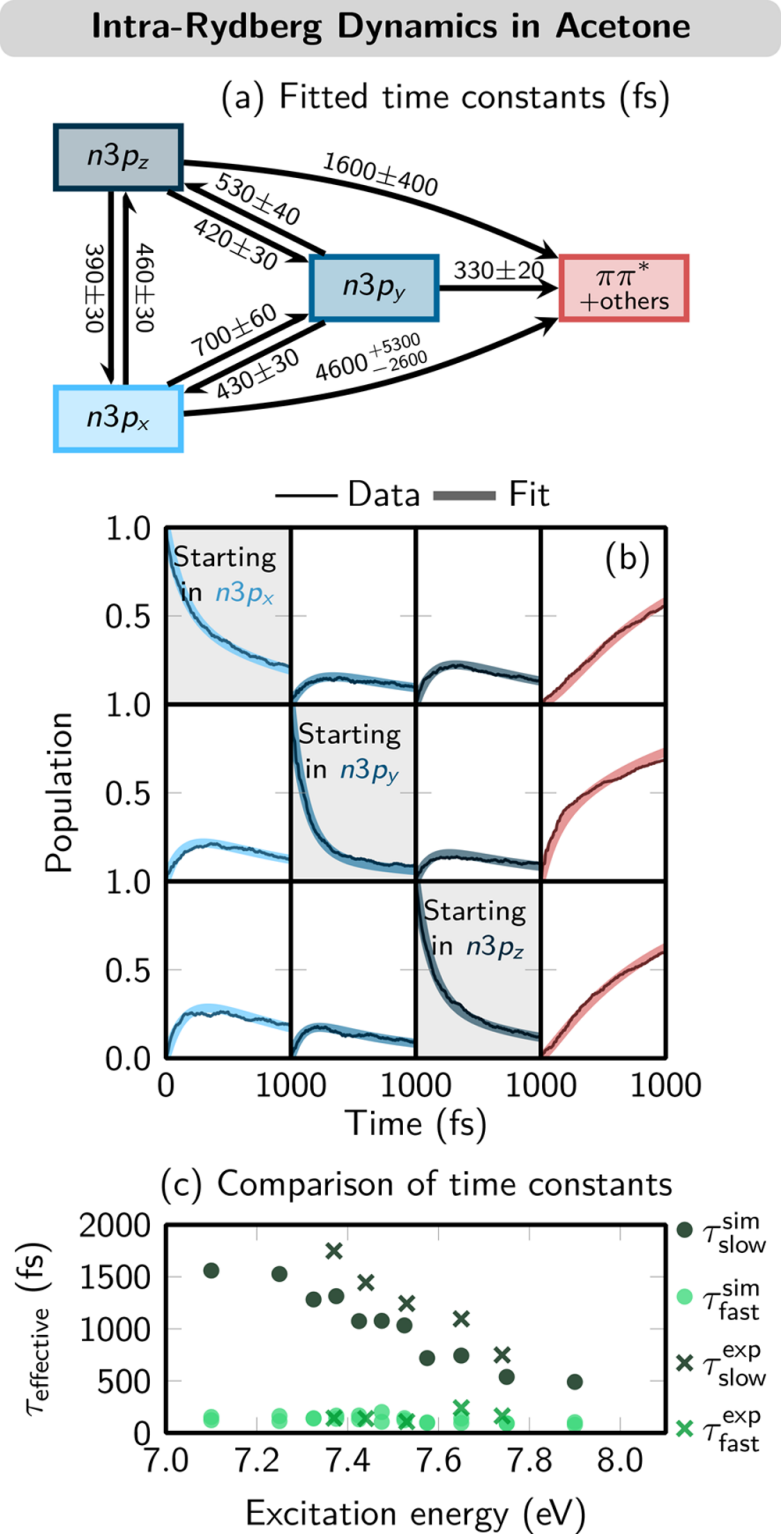
(a) Kinetic model and globally fitted time constants obtained from the population data for acetone. (b) Evolution of diabatic populations after excitation to different n3p Rydberg states. (c) Comparison of the effective time constants from simulation and time-resolved photoelectron spectroscopy. Adapted from ref (40). Copyright 2020 American Chemical Society.

Acetone was studied in the gas phase with SH/LVC in order to resolve the population dynamics among the set of the three near-degenerate n3p Rydberg states.^40^ The main challenge stems from the many different internal conversion processes (n3p_*x*_ ⇄ n3p_*y*_ ⇄ n3p_*z*_, n3p_*x*_/n3p_*y*_/n3p_*z*_ → ππ*) that take place simultaneously (Figure 3a), preventing one from obtaining all of the involved time constants from experimental data, e.g., from time-resolved photoelectron spectroscopy.

The population dynamics was investigated using an LVC model with all 24 DOFs and 49 diabatic states fitted with spin-opposite-scaled algebraic diagrammatic construction to second order [SOS-ADC(2)].^40^ We simulated three independent ensembles of roughly 1000 trajectories each, starting in the three different n3p Rydberg states. After excitation, non-adiabatic transitions quickly equilibrate the populations of the three Rydberg states while the population more slowly decays to the lower-lying ππ* state via the n3p_*y*_ state. A global fit of the kinetic model to all of the data (Figure 3b) provided nine time constants to be compared with experimental data. The agreement between the experimental and effective theoretical time constants (Figure 3c), including the excitation energy dependence of the time constants, is very good, demonstrating the usefulness of SH/LVC at a very small expense (ca. 600 CPUh for 3 ns of simulation that otherwise would have cost about 3 000 000 CPUh).

### 3.3 Iron(II) NHC Photosensitizer

**Figure 4:**
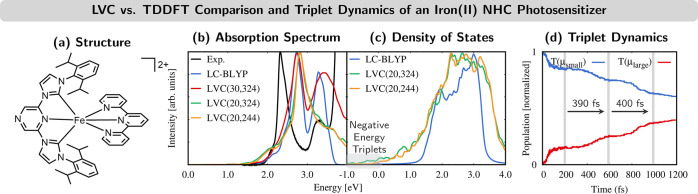
(a) Structure of [Fe^II^(tpy)(pyz-NHC)]^2+^ (tpy = 2,2′:6′,2″-terpyridine; pyz-NHC = 1,1′-bis(2,6-diisopropylphenyl)pyrazinyldiimidazolium-2,2′-diylidene). (b) Experimental absorption spectrum and calculated absorption spectra from LC-BLYP and LVC(*N*,*M*) models (*N* = number of electronic states, M = number of normal modes). (c) Triplet density of states. (d) Time evolution of triplet-state populations. Adapted from ref (41). Copyright 2020 American Chemical Society.

Because of their large size, performing nuclear dynamics in transition metal complexes is intimidating. Here we show how using the SH/LVC approach enabled simulation of the deactivation dynamics of [Fe^II^(tpy)(pyz-NHC)]^2+^ (see Figure 4a), an iron-based photosensitizer featuring a N-heterocyclic carbene (NHC) ligand.^41^ Strongly σ-donating ligands such as NHCs can destabilize low-lying metal-centered states in 3d metal complexes to enable long-lived metal-to-ligand charge transfer (MLCT) states to be harnessed for dye-sensitized solar cell applications.^44^

In [Fe^II^(tpy)(pyz-NHC)]^2+^, the LVC model was parametrized from a time-dependent density functional theory (TDDFT) calculation using an optimally tuned LC-BLYP functional.^41^ This level of theory reproduces the experimental absorption spectrum (blue curve vs black curve in Figure 4b). This agreement is also maintained when an LVC model including 30 singlet electronic states is used (red curve). A reduced LVC model with 20 states (green curve) is also sufficient to describe the first absorption band and the dynamics initiated from this band. However, although it is not visible in the absorption spectrum, this LVC model has problems in describing the triplet states. As shown in Figure 4c, compared with the triplet density of states (DOS) (computed from a Wigner distribution) of the LC-BLYP reference (blue curve), the LVC model (green curve) predicts triplet DOS at very low energies. Some DOS even appeared at negative energies (gray box), which leads to spurious S_0_ → T_1_ ISC of trajectories that had already relaxed to S_0_. This problem was alleviated by removing those normal modes that contributed most to the appearance of the low-energy triplet DOS: low-frequency modes related to torsional motions and vibrations in the molecular backbone.^41^ Keeping 244 modes (out of 324) eliminated the spurious negative-energy triplets (orange curve in Figure 4c) and thereby the nonphysical S_0_ → T_1_ ISC while leaving the absorption spectrum mostly unaffected (orange curve in Figure 4b).

Using the truncated LVC model in SHARC up to 2 ps, we found that after excitation to the singlet manifold and ultrafast ISC in the first 50 fs, mostly triplet states with small dipole moment were populated. Interestingly, a stepwise population decay to triplets with larger dipole moment with a period of about 400 fs was observed (see Figure 4d). Similar oscillatory signals had been observed previously in X-ray absorption^45^ and emission^46^ experiments on related iron(II) complexes.

### 3.4 Disulfide/Dithiol Ruthenium(II) Photoswitch

**Figure 5:**
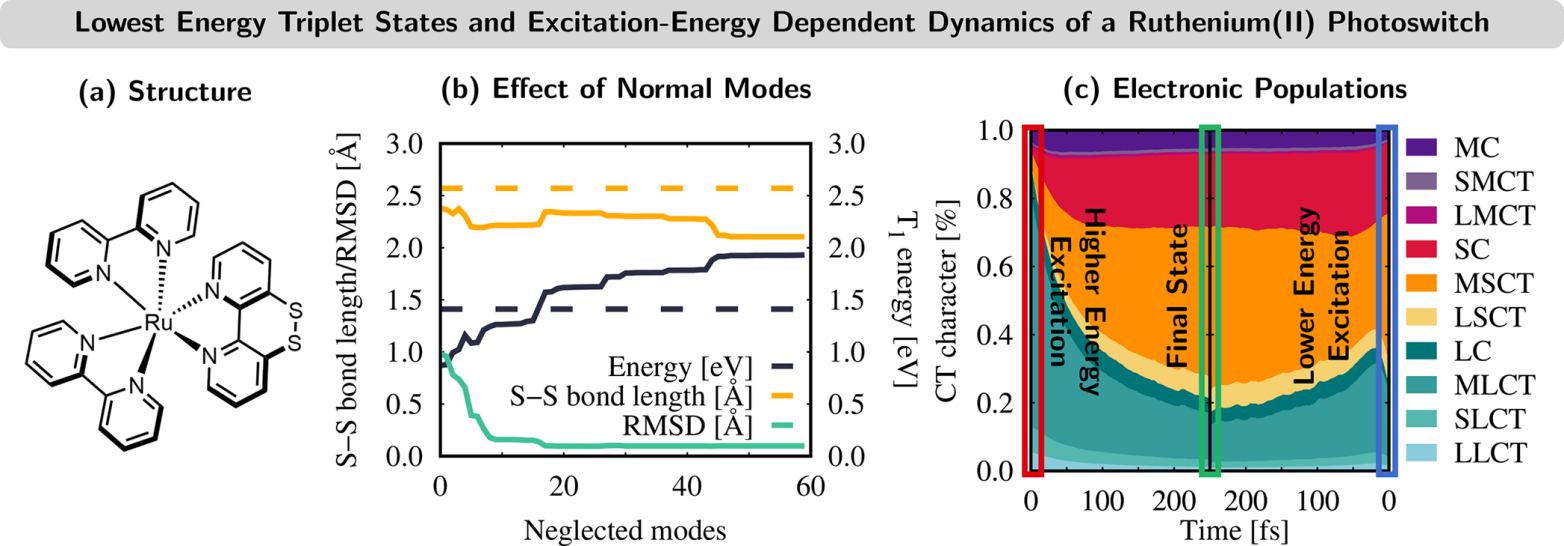
(a) Structure of [Ru^II^(^SS^bpy)(bpy)_2_]^2+^ (bpy = 2,2′-bipyridine; ^SS^bpy = [1,2]dithiino[4,3-*b*:5,6-*b*′]dipyridine). (b) Effect of removing normal modes on the T_1,min_ energy, S–S bond length, and RMSD of all nuclear coordinates. LVC (B3LYP) results are shown as solid (dashed) lines. (c) Time evolution of the electronic-state populations for two different excitation energies. Different colors correspond to different charge-transfer contributions:^42^ M = ruthenium, S = ^SS^bpy, L = bpy. Excitations within each fragment are denoted as MC, SC, and LC. Charge transfer between fragments is denoted by XYCT, whereby an electron is excited from X to Y. Adapted from ref (43). Copyright 2021 American Chemical Society.

Here we show another example of transition metal photodynamics, this time for [Ru^II^(^SS^bpy)(bpy)_2_]^2+^ (Figure 5a). The LVC model was parametrized with the B3LYP functional.^43^ This complex features a modified bipyridine ligand with a dithiol/disulfide switch that can be potentially utilized in multiredox reactions via photoactivated proton-coupled electron transfer.^47,48^

As in the previous Fe complex, the full 177-dimensional LVC model was problematic. Both TDDFT and the corresponding LVC model describe the absorption spectrum well,^43^ but the LVC model could not properly reproduce the geometry of the lowest-energy triplet state (T_1_) minimum—the final state reached in the dynamics. With TDDFT, the T_1_ geometry has planar bpy pyridine rings and a S–S bond length of 2.57 Å. In contrast, the LVC model predicts twisted bpy units and a S–S bond length of only 2.43 Å. Furthermore, the T_1_ minimum is found at an energy of 0.9 eV above the S_0_ minimum, which is much lower than the value of 1.4 eV given by TDDFT. Accordingly, normal modes were removed from the LVC model. Figure 5b illustrates the effect that eliminating problematic modes has on the T_1_ geometry (green curve), the S–S bond length (orange curve), and the T_1,min_ energy (black curve). Although it is not possible to reach the exact S–S bond lengths, removing the 16 most problematic modes—mostly low-frequency modes—leads to a T_1,min_ energy of 1.57 eV (RMSD of only 0.09 Å) and a S–S bond length of 2.34 Å, in satisfactory agreement with TDDFT.

The resulting 161-dimensional LVC model was employed in two sets of dynamical simulations starting at different excitation energies that correspond to different electronic states in the absorption spectrum. The higher energies initially populate MLCT states with charge transfer from Ru to the bpy ligands (green contributions in the red box in Figure 5c). The lower energies populate MLCT states with charge transfer from Ru to the ^SS^bpy ligand (orange/red contributions in the blue box). The simulations beautifully show that regardless of the initial energy, after 250 fs (green box) states involving MLCT to the ^SS^bpy ligand are populated.

### 3.5 Vanadium(III) Near-Infrared Luminophore

**Figure 6:**
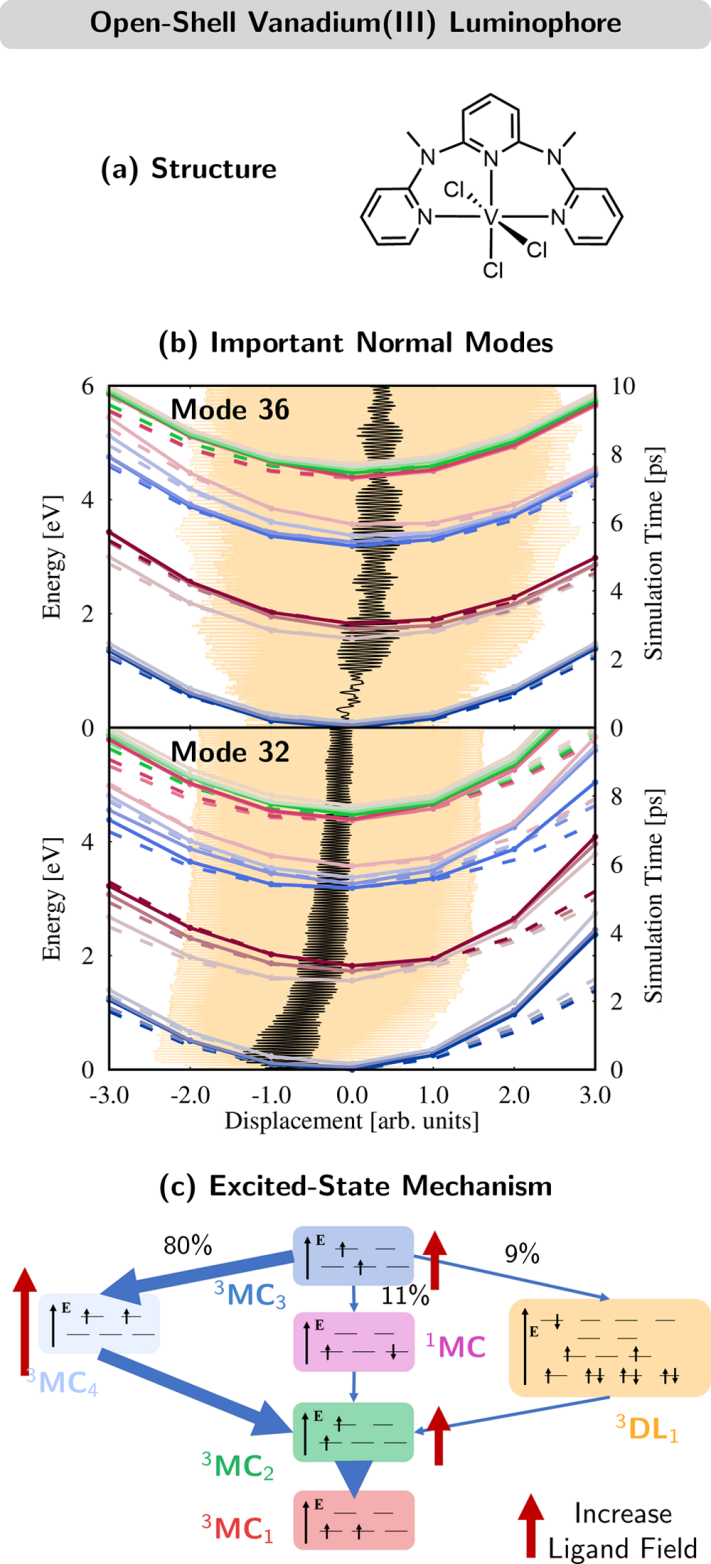
(a) Structure of V^III^(Cl)_3_(ddpd) (ddpd = *N*,*N*′-dimethyl-*N*,*N*′-dipyridin-2-ylpyridine-2,6-diamine). (b) Scans comparing LVC (dashed lines) and CASSCF (solid lines) potentials (in eV, left axis) of important normal modes and the time evolution of its average (black curve) and standard deviation (orange areas) (in ps, right axis). (c) Excited-state mechanism obtained from the SH/LVC dynamics. Red arrows indicate the energetic shifts of the states resulting from an increase in the strength of the ligand field. Adapted from ref (2). CC BY 3.0.

An interesting challenge for excited-state dynamics simulations is posed by open-shell complexes, like the near-infrared-emitting complex V^III^(Cl)_3_(ddpd) shown in Figure 6a.^2^ V^III^(Cl)_3_(ddpd) features a vanadium d^2^ electron configuration in the ground state, producing three near-degenerate triplet states (with nine spin components in total), which calls for a multiconfigurational treatment.

Accordingly, the LVC model was parametrized at the CASSCF level of theory with a (10,13) active space including five vanadium d orbitals and eight ligand π/π* orbitals. The parametrization of the LVC model including 15 singlet states, 16 triplet states, and 123 normal modes was one of the most expensive we performed, demanding about 40 000 CPUh. This full model was used without difficulties to propagate 2000 trajectories for 10 ps, costing another 60 000 CPUh.

Figure 6b exemplifies the general good agreement of the reference CASSCF PESs and the LVC model. For the important mode 32, compliance worsens for large positive displacements; luckily, this region is never visited by the trajectories (orange-shaded area). The resulting excited-state mechanism is plotted in Figure 6c. Starting from the ^3^MC_3_ state (dark blue), the major deactivation channel is internal conversion via the doubly excited ^3^MC_4_ state (light blue). ISC to the ^1^MC states (violet)—which is responsible for the near-infrared luminescence—is only a minor channel. The analysis of the trajectories suggests that introducing ligands with increased ligand-field splitting should destabilize states with electrons in the antibonding e_g_^*^ orbitals, such that the internal conversion pathway through ^3^MC_4_ and the decay of ^1^MC to ^3^MC_2_ are quenched. Both effects should then enhance the population of the emissive ^1^MC state, which is less affected by an increased ligand field.

### 3.6 Ultrafast Charge Separation in Re(I)-Sensitized Azurin

**Figure 7:**
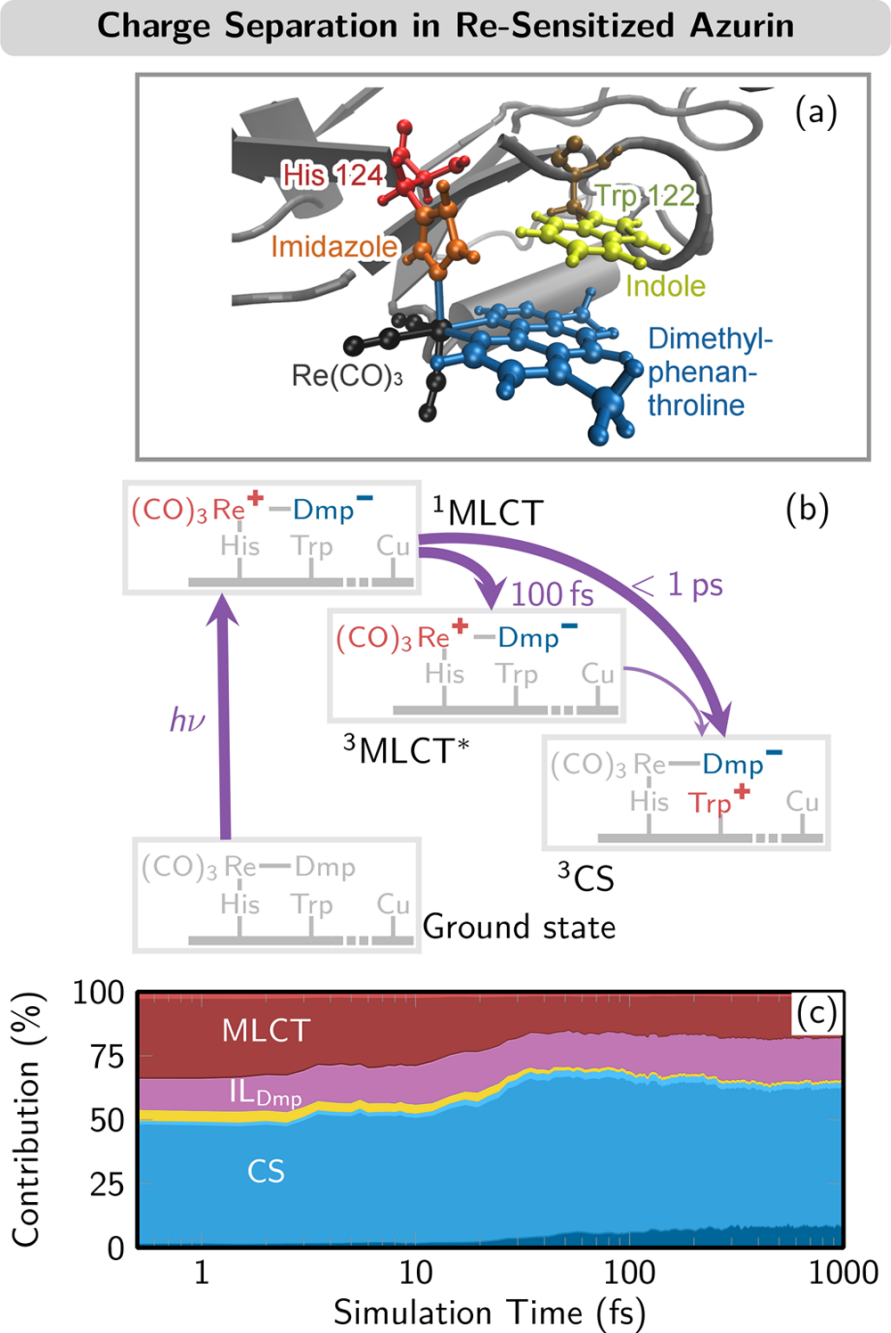
(a) Structure of the [Re(CO)_3_(dmp)]^+^ photosensitizer (dmp = 4,7-dimethylphenanthroline) attached to azurin close to the indole ring of tryptophan 122. (b) Scheme of charge transfer states after photoexcitation. (c) Temporal evolution of the electronic wave function character, with the largest contributions MLCT, IL_Dmp_ (intraligand excitation on dimethylphenanthroline), and CS (charge-separated, i.e., charge transfer from tryptophan to dimethylphenanthroline). Adapted from ref (29). CC BY 4.0.

In this last example,^29^ we illustrate how SH/LVC can handle charge transfer in a biological donor–acceptor system. [Re(CO)_3_(dmp)]^+^ is attached near a tryptophan in the small protein *Pseudomonas aeruginosa* azurin^49^ (Figure 7a). This is a challenging case for LVC because systems with multiple fragments might be too flexible to describe with linear normal modes—however, the protein framework gives the system sufficient rigidity. To create the LVC model, the protein was replaced by two methyl groups that were fixed in space for the normal mode computation. Subsequently, the model was parametrized from TDDFT calculations with the B3LYP functional, and as in the examples in sections 3.3 and 3.4, it had to be reduced from 192 to 158 normal modes and from 30 singlets and 30 triplets to 20 singlets and 17 triplets to avoid spurious low-energy states.

The charge transfer dynamics of this system involves MLCT states—i.e., excitations from Re to dmp—and excitations from tryptophan to dmp, called charge-separated (CS) states. Experimental studies hypothesized^49^ that photoexcitation produces exclusively singlet MLCT states, which subsequently evolve to triplet MLCT states via ISC and subsequently or in parallel to CS states via electron transfer from tryptophan to Re (see Figure 7b). However, our SH/LVC simulations predicted that in fact the CS states are already populated during absorption (Figure 7c, left) given their nonvanishing transition moments. The trajectories also showed that the first picosecond of dynamics is dominated by extensive back-and-forth transfer between MLCT and CS states, slowly shifting towards an increased CS contribution (Figure 7c, right). We note here that these useful CT analyses (cf. also Figures 5c and Figure 6c) are extremely easy to obtain within SH/LVC because of the availability of a well-defined adiabatic-to-diabatic transformation matrix.

## 4 Validation of Surface Hopping Using LVC Models

### 4.1 Testing the Limits of Surface Hopping

SH has become popular thanks to its conceptual simplicity and ease of interpretation. However, its simplicity is deceptive, as the classical simulations still need to describe a number of critical quantum phenomena. These include (i) coherence and eventual decoherence of the wave packet after it branches onto different electronic states, (ii) exchange of energy and momentum between electronic and nuclear degrees of freedom, and (iii) the possibility that the quantum wave packet will undergo classically forbidden electronic transitions. To overcome these challenges, SH simulations usually employ ad hoc corrections for (i) quantum decoherence, (ii) momentum rescaling after surface hops, and (iii) classically forbidden “frustrated” hops.

Different choices for (i)–(iii) demand testing against a QD reference. Traditionally, low-dimensional model systems were used with that aim, even if it is unclear how well those models reflect realistic dynamics. Alternatively, SH on ab initio potentials was compared to QD using model potentials, but the use of different PESs severely questions the comparability. Here we argue that SH/VC might be the ideal test bed, as the same realistic multidimensional PESs can be used consistently in both SH and QD.

With independent trajectories, no rigorous solution exists for computing decoherence (i), but a number of correction schemes have been devised, such as the energy-based decoherence (EDC) correction^50,51^ and the augmented fewest switches SH (AFSSH) algorithm.^52^ During a surface hop, the nuclear momentum vector is usually adjusted (ii), for which also different approaches exist. Often the entire vector is rescaled to conserve the total energy (*E*), or alternatively, one may not adjust anything to trivially conserve the nuclear momentum (**p**). Both approaches benefit from the fact that no other properties need to be calculated. However, adjusting the entire vector leads to a rather even distribution of changes in the kinetic energy across the system, neglecting the participation of specific bonds and movements, while both approaches may result in a violation of detailed balance. More sophisticated approaches aim at conserving both energy and momentum by adjusting the momentum along only one direction: along either the gradient difference vector (*E***p**_**g**_) or the NAC vector (*E***p**_**h**_). The latter is considered the highest-level method, which can be related to exact dynamics.^23,53^ Especially with *E***p**_**g**_ or *E***p**_**h**_, often during hops to a higher-lying PES not enough kinetic energy (or linear momentum) is available in the relevant direction. During these “frustrated hops” (iii), typically either nothing is done (denoted “*+*”) or the trajectory is reflected along the **g** or **h** vector (“–”) under certain conditions.^54^

**Figure 8:**
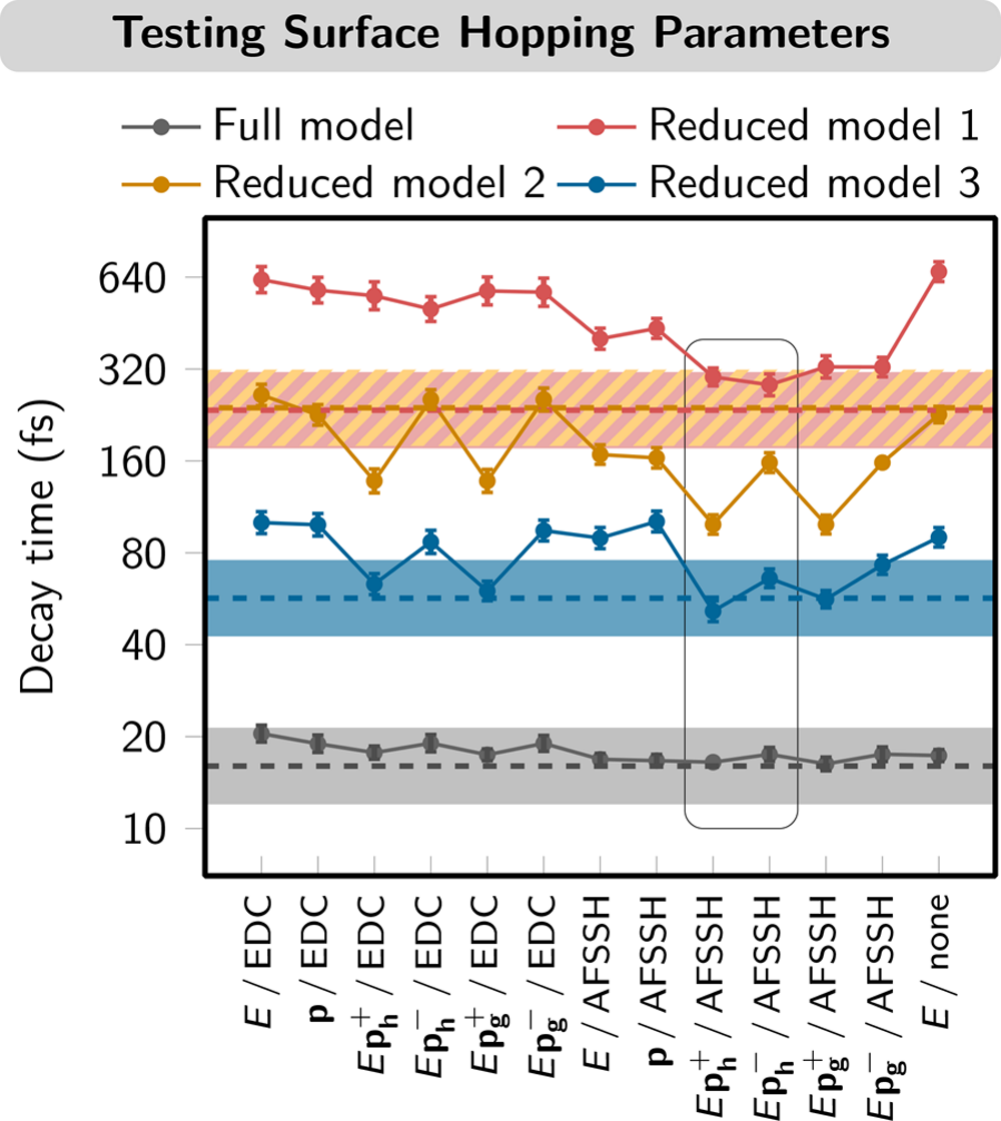
Comparison of decay time constants obtained for four different LVC models of [Re(CO)_3_(im)(phen)]^+^ (im = imidazole; phen = phenanthroline). Fitted decay time constants from MCTDH/LVC are shown as dashed lines (shaded areas indicate acceptable error intervals). It should be noted that the results of models 1 and 2 deliver almost identical decay times and errors (brown/orange superimposed dashed lines and hatched pattern). The fitted decay times and standard errors of SH/LVC simulations with 13 different settings (see the text) are given as colored dots and error bars, respectively. The best-performing protocols are indicated by the box. Data were taken from ref (3).

Combining the above options for treating issues (i)–(iii), we generated 13 combinations of individual SH protocols (see the bottom of Figure 8).^3^ It should be noted that not all of these options are actually well-advised to use from both a theoretical and practical perspective.^55,56^ With the 13 protocols, we benchmarked ISC time scales in [Re(CO)_3_(im)(phen)]^+^ using SH against MCTDH based on previously available^57^ LVC potentials. As the model contains a dense manifold of states requiring many hops between them, it constituted a challenging case for SH dynamics. We used a full-dimensional LVC model and three reduced LVC models, on which an ensemble of 200 trajectories were propagated for each of the 13 protocols. In total, the data set contained more than 10 000 trajectories and 10 000 000 formal single-point computations–an impractical endeavor for standard on-the-fly SH.

The resulting fitted ISC decay times are displayed in Figure 8. Encouragingly, all of the protocols reproduced the correct order of magnitude of the time scales; however, none was in fact able to place all of the data points on top of the MCTDH reference data (dashed lines in Figure 8). Most striking is the realization that the differences among protocols seem quite erratic, with minor adjustments causing notable changes. Previous comparisons of the performance of various SH protocols focused on analyzing the influence of a single effect,^55,56^ but the LVC model opens up the possibility of investigating combinations and synergies between different protocols. In the presented publication, the best-performing protocols are AFSSH/*E***p**_**h**_^+^ and AFSSH/*E***p**_**h**_^–^ (box in Figure 8), which place at least three of the four time scales within the error bar.

### 4.2 Automated Reduction of Dimensionality

Using MCTDH to compare to SH has one bottleneck: the cost of the MCTDH calculation. For that reason, usually MCTDH is restricted to a subset of nuclear degrees of freedom and a small number of electronic states.^5,58−60^ Being presented with the opportunity to combine the best of both worlds—the accuracy of QD and the computational simplicity of SH—in 2019 we proposed an approach that could benefit both parties: the *SHARC-gym*.^4^ Metaphorically speaking, the SHARC-gym is an iterative procedure that allows a system to *lose weight*, i.e., to determine which nuclear and electronic DOFs can be spared.

**Figure 9:**
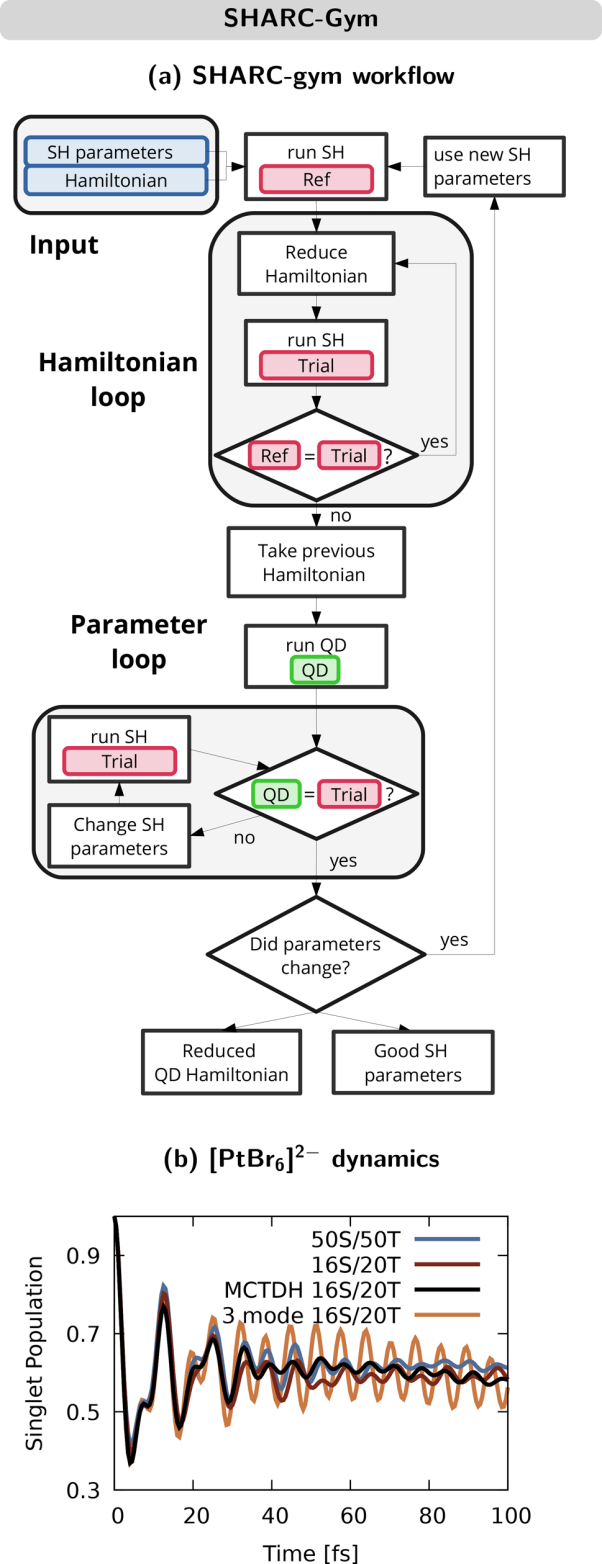
(a) Schematic representation of the SHARC-gym workflow, including SH and QD steps. Adapted from ref (4). Copyright 2019 American Chemical Society. (b) Populations in all singlet states of [PtBr_6_]^2–^ in the SH and MCTDH simulations, featuring different numbers of singlet (S) and triplet (T) excited states and modes. Data were taken from ref (4).

The SHARC-gym is built on three pillars^4^ (see Figure 9a): a reference SH dynamics (*Input*), a loop to remove unimportant normal modes and electronic states (*Hamiltonian loop*), and a second loop that validates the SH protocol against MCTDH (*Parameter loop*). Once the iterative SHARC-gym finishes, one obtains an optimized (QD-validated) SH protocol and a reduced LVC Hamiltonian that is able to capture the full-dimensional dynamics.

This methodology was first applied to [PtBr_6_]^2–^. As a start, a 15-mode LVC Hamiltonian spanning 50 singlet states and 50 triplet states from a B3LYP reference was parametrized. The first iteration of the SHARC-gym reduced the Hamiltonian to six singlet states and 11 triplet states, but a comparison with MCTDH dynamics showed that the initial SH setup was not suitable to describe the ISC dynamics of [PtBr_6_]^2–^. Contrary to other cases,^1^ here the EDC^51^ was in worse agreement with the MCTDH reference than using no decoherence correction at all, showcasing that the adequacy of these corrections depends on the system. Repeating the SHARC-gym with a refined SH protocol (no decoherence) and a Hamiltonian with 16 singlet states and 20 triplet states led to an excellent agreement of SH with MCTDH dynamics (see Figure 9b). Then, this Hamiltonian was used to reduce the nuclear DOFs based on the sum of κ_*i*_^(*n*)^ and λ_*i*_^(*n,m*)^ terms for each mode (eq 4). Finally, we found that the essential dynamics of [PtBr_6_]^2–^ can be captured using merely three modes: the totally symmetric mode and a doubly degenerate e_g_ mode pair that feature the strongest Jahn–Teller couplings.

### 4.3 Inclusion of External Fields

Having an approach that allows testing of the limitations of SH and comparing SH against MCTDH is ideal to investigate the suitability of SH in the presence of explicit laser pulses.^61^ To that aim, we used two molecules: SO_2_ and the larger 2-thiocytosine. The SHARC-gym was employed to reduce 2-thiocytosine to an LVC Hamiltonian with only 10 modes, making it affordable in MCTDH. For each molecule, 36 SH protocols were evaluated in both the absence and the presence of an external field with various pulse lengths. Although no ideal set of SH parameters was found, clear trends between protocols that performed well and those that performed poorly were identified. Our study additionally revealed incompatibilities between certain SH correction schemes that, when paired together, performed exceptionally poorly. For example, for long laser pulses, interference effects, which are poorly modeled in SH, became particularly important. The more vibrational modes are involved, the less important is interference, as the dephasing of the wave packet increases, opening up different deactivation channels.

This extensive benchmark required more than 200 000 trajectories, accumulating more than 900 000 000 single point evaluations—a feat unobtainable without the efficient SH/LVC model and the pysharc implementation. The low cost of the SH/LVC setup has an additional advantage in light-induced simulations, where it is a common issue that a considerable number of trajectories are not excited by the external field and therefore do not provide information on the excited-state dynamics but bear high computational cost. SH/LVC softens this blow by decreasing the overall computational cost.

## 5 Conclusion and Perspective

**Figure 10:**
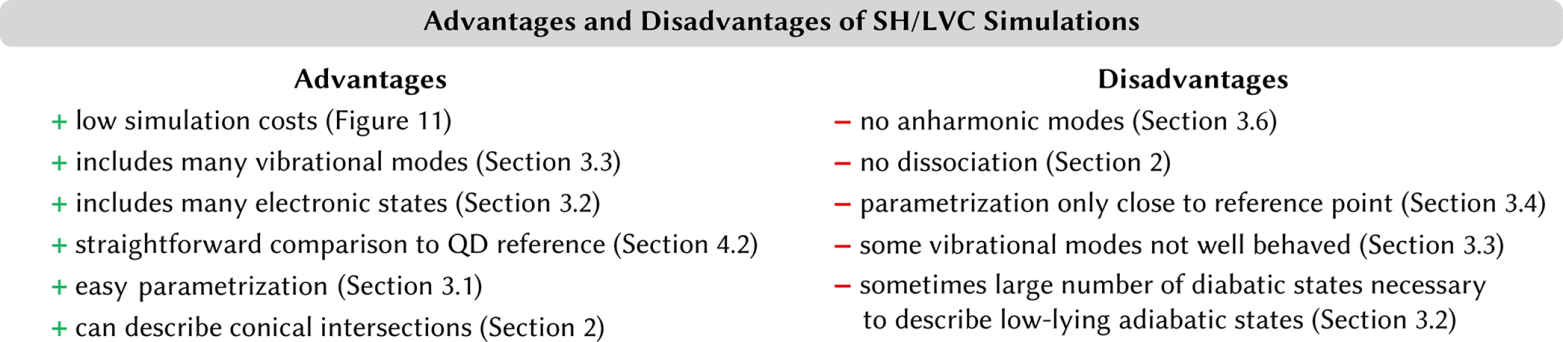
List of advantages and disadvantages of SH simulations using LVC models.

In this Account, we have presented the applicability of vibronic coupling models combined with surface hopping trajectory methods. We argue that this approach has great potential to perform highly efficient non-adiabatic molecular dynamics simulations that include a large number of nuclear and electronic DOFs for extended simulation times on large ensembles of trajectories. The superior efficiency of SH/LVC not only allows computer simulations of systems of different complexity but also enables rigorous testing of diverse SH parameters introduced to recover quantum effects that until now could be investigated only with low-dimensional model systems. The advantages of SH/LVC simulations alongside possible disadvantages are summarized in Figure 10, where we have added references to sections within this review, which can guide further reading.

**Figure 11:**
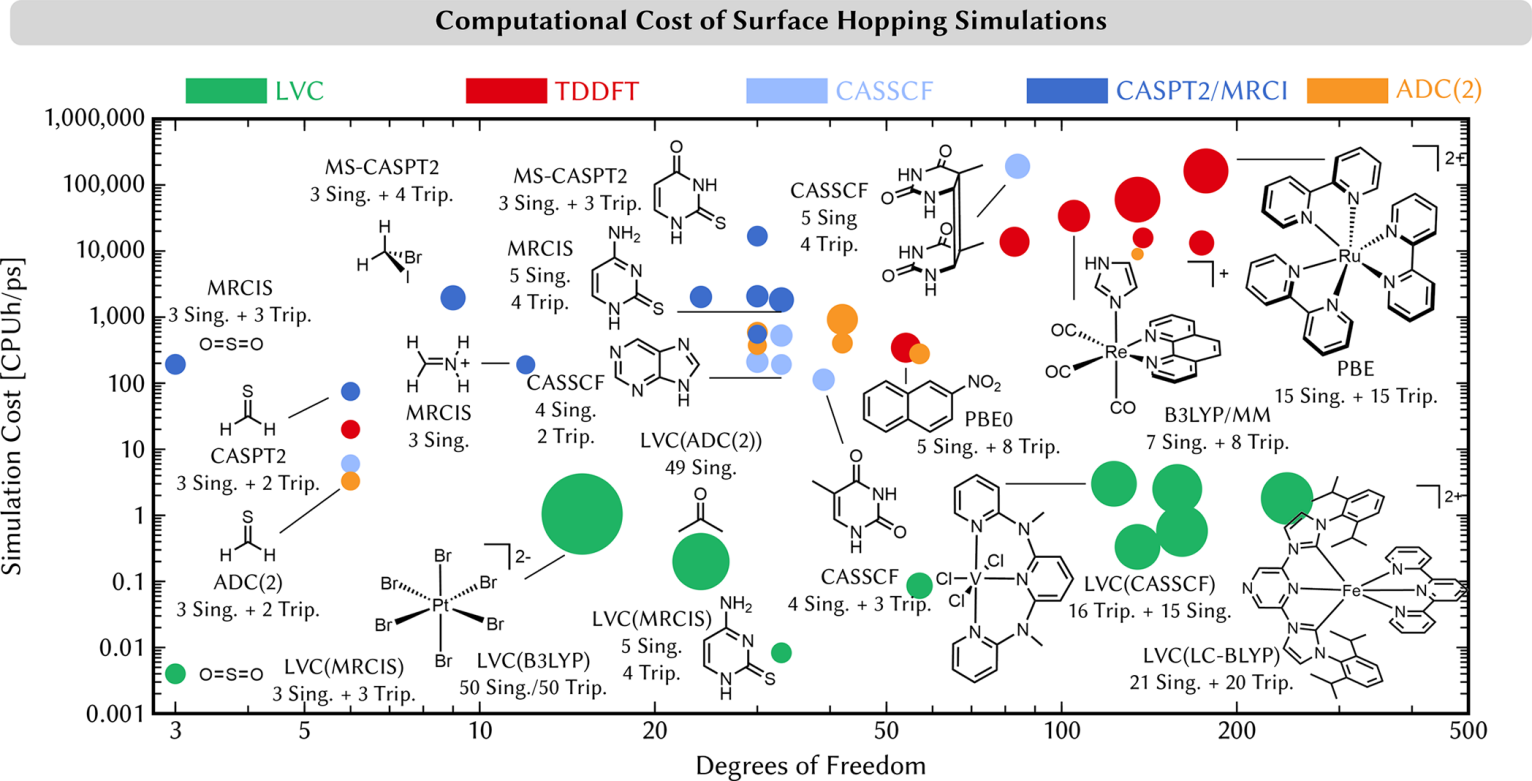
Computational cost in CPUh per propagated picosecond (of a single trajectory) for SH simulations as a function of the nuclear degrees of freedom using LVC and different on-the-fly electronic structure methods. The double logarithmic scale should be noted. The areas of the circles are proportional to the numbers of electronic states included. MM indicates that the solvent was described by molecular mechanics. The computational costs are only qualitative, as they were obtained from different electronic structure codes and computer architectures. The simulations were performed between 2013 and 2021.

To highlight the performance of SH, we have collected the computational costs of different SH simulations conducted in our lab in the past decade using either on-the-fly SH or SH/LVC (Figure 11). The cost of the on-the-fly SH simulations is clearly correlated with the expense of the underlying quantum-chemical calculations: even at the pace at which quantum chemistry has developed in the past decade, the dynamics of the largest systems could be studied only with TDDFT. In comparison, the computational cost of SH/LVC simulations is several orders of magnitude lower. Most LVC examples cluster around 1 CPUh per propagated picosecond and include up to a few hundred nuclear DOFs and dozens of electronic states.

One can expect the saved time to be reinvested in obtaining PESs at higher levels of theory or extending LVC to second^35,36^ or higher^62,63^ orders that could possibly avoid the troubles encountered by some of the LVC Hamiltonians revisited here. Indeed, QVC models (recall eq 5) incorporate quadratic diagonal coupling terms related to the frequency shifts of the states, bilinear diagonal couplings related to Duschinsky rotations (effectively introducing different normal modes for different states), and second-order off-diagonal couplings that modify the couplings between the electronic states—all providing further flexibility to the PESs. Other conceivable PES improvements include anharmonic diabatic Morse potentials,^64−66^ state-specific quartic functions,^64^ or merging with specifically designed potentials (e.g., unbounded potentials^66,67^) to describe dissociation or trigonometric functions^65,68,69^ to account for torsional motion. We expect this Account to stimulate work along these lines and empower further full-dimensional dynamics simulations of increasingly large molecules for long times, contributing to a better understanding of photochemical processes.

## Abbreviations

AFSSH: augmented fewest-switches surface hopping
CASSCF: complete-active-space self-consistent field
CPUh: core hour (unit of computation time)
CS: charge-separated
CT: charge transfer
DOF: degree of freecom
DOS: density of states
EDC: energy-based decoherence correction
ISC: intersystem crossing
LC: ligand-centered
LVC: linear vibronic coupling
MC: metal-centered
MCTDH: multiconfigurational time-dependent Hartree
MLCT: metal-to-ligand charge transfer
MRCIS: multireference configuration interaction including single excitations
NAC: non-adiabatic coupling
NHC: N-heterocyclic carbene
PES: potential energy surface
QD: quantum dynamics
QVC: quadratic vibronic coupling
RMSD: root-mean-square displacement
SH: surface hopping
SHARC: surface hopping including arbitrary couplings
SOS-ADC(2): spin-opposite-scaled second-order algebraic diagrammatic construction
TDDFT: time-dependent density functional theory
VC: vibronic coupling
